# On the origin of distribution patterns of motifs in biological networks

**DOI:** 10.1186/1752-0509-2-73

**Published:** 2008-08-12

**Authors:** Arun S Konagurthu, Arthur M Lesk

**Affiliations:** 1 The Huck Institute for Genomics, Proteomics, and Bioinformatics, Department of Biochemistry and Molecular Biology, The Pennsylvania State University, University Park, PA 16802, USA

## Abstract

**Background:**

Inventories of small subgraphs in biological networks have identified commonly-recurring patterns, called motifs. The inference that these motifs have been selected for function rests on the idea that their occurrences are significantly more frequent than random.

**Results:**

Our analysis of several large biological networks suggests, in contrast, that the frequencies of appearance of common subgraphs are similar in natural and corresponding random networks.

**Conclusion:**

Indeed, certain topological features of biological networks give rise naturally to the common appearance of the motifs. We therefore question whether frequencies of occurrences are reasonable evidence that the structures of motifs have been selected for their functional contribution to the operation of networks.

## Background

The network or directed graph description has become the preferred representation of the integrated activity of components of biological processes. The exponential growth of biological network data in the last five years has its source in recent advances in technologies such as mass spectrometry, genome-scale ChiP-chip experiments, yeast two-hybrid assays, combinatorial reverse genetic screens, and rapid literature mining techniques [1].

The science of systems biology has the aim of understanding the functional constraints and design principles of biological networks. Alon and colleagues were the first to introduce the notion of "motifs" in biological networks [2,3]. Motifs are small patterns observed to recur throughout a network, with frequencies statistically higher than expected in random networks of similar connectivity parameters. Since the introduction of this concept, motifs have been reported in many biological networks: metabolic, signaling pathway, protein-protein interaction, and ecological networks amongst others [2-6]. Moreover, the prevalence of motifs is often considered as evidence for evolutionary selection, for implementing a *specific *function [2,3,7]. Motifs are believed to be building blocks of the functional architecture of a biological network [3].

**Figure 1:**
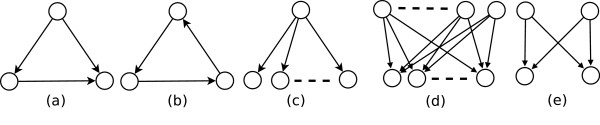
**Canonical subgraph patterns in biological networks**. Canonical subgraph patterns in biological networks. (a) Feed-forward loop (FFL): contains a "source" (at the top), "intermediate" (bottom-left), and "target" (bottom-right) nodes. (b) 3-cycle: a three node directed cyclic graph, (c) Single-input module (SIM). (d) Multiple-input module (MIM). (e) Bifan motif. SIM, MIM, and Bifan are two-layered graphs with edges from nodes in top- to bottom-layer. A Bifan is a MIM with exactly 2 parent and 2 child nodes.

Consider for example the canonical set of motifs in transcription regulatory networks: Single input module (SIM), Multiple input module (MIM), and Feedforward loop (FFL) [3]. (See Figure 1. Originally, Alon and colleagues [2] proposed a *dense overlapping regulon *(DOR) as a motif; MIMs are special DORs that arose as a generalization of Bifan motif). Specific functions have been ascribed to each type of motif [2,7-11]: SIMs are commonly associated with temporal ordering of gene expression, MIMs with combinatorial gene regulation, and FFLs with filters that do not pass on transient signals [2]. These functions depend not only on the topology of the subgraph, but on the logic at nodes receiving multiple inputs. The common occurrence of these motifs, relative to corresponding randomized graphs, has been taken as evidence for their selection for function.

In this paper we investigate the role of small network subgraphs as building blocks of biological networks. We analysed several biological networks: transcription regulation networks of *Saccharomyces cerevisiae *under different physiological conditions, the transcription regulation network of *Escherichia coli*, and a neuronal signalling pathway network of the hippocampal CA1 neuron.

Contrary to previous reports, we find that commonly accepted motifs are neither over- nor under-represented in these real networks in comparison to their random formulations. We discuss how the topology of biological networks automatically predisposes them to contain a certain distribution of motifs. This suggests that the evidence for the functional significance of motifs should be reevaluated.

## Methods

We use the transcription regulatory networks of *Saccharomyces cerevisiae *under various physiological conditions – composite, cell cycle, sporulation, diauxic shift, DNA damage, and stress response – published by Luscombe and coworkers [5]. Their largest (composite) network contains 3459 nodes and 7014 interactions ().

To aid comparison of our work with that of Shen-Orr et al. [2], we also use their *Escherichia coli *transcription network containing 424 nodes and 577 interactions ().

Additionally, we use the neuronal signalling pathway network of the hippocampal CA1 neuron published by Máayan and colleagues, containing 594 nodes and 1422 interactions [6] ().

We implemented Ullmann's algorithm for subgraph isomorphism [12] to enumerate fixed sized subgraph patterns (*e.g*. FFL, 3-cycle).

In enumerating variable sized (maximal) subgraph patterns such as SIMs and MIMs, we used our algorithms described in [13]. We note that Bifans are counted as MIMs with *exactly *two elements each in both parent and child sets. (See Definitions.)

To generate random networks conserving the degree sequence of the real network, we use the method described by Shen-Orr et al. [2]: Starting with the same number of nodes as in an original network, nodes in the random graph are assigned a specific number of in- and out-"edge-stubs." Randomly chosen pairs of in- and out-edge-stubs are joined, giving rise to a random (directed) graph.

### Definitions

A FFL is a set of three nodes (source, intermediate, and target) with one direct path, and another indirect path through an intermediate node, from source to target (See Figure 1(a)).

A 3-cycle (3-CYC) is a three-node directed cyclic graph (Figure 1(b)).

Single and multiple input modules (SIM and MIM) in a directed graph are *maximal *subgraphs comprising two non-empty disjoint sets (layers): P and C (standing for Parent and Child). By maximal we mean, for example, that each MIM is not contained in a larger MIM.

A SIM requires that C contain only one node and C contain at least two nodes, such that the full graph contains an edge from the parent node to every *c*_*i *_∈ C. We also require that the *indegree *– number of incoming edges – of every *c*_*i *_to be strictly equal to one: within the full network, not just within the subgraph. By this definition of a SIM, no edges can exist between any *c*_*i*_, *c*_*j *_∈ C. It follows that P is the only parent of all nodes in set C.

A MIM requires that both P and C must contain ≥ 2 nodes, that there is an edge from every *p*_*i *_∈ P to every *c*_*i *_∈ C, no edge between any *p*_*i*_, *p*_*j *_∈ P, and no edge between any *c*_*i*_, *c*_*j *_∈ C. A Bifan is a *maximal *MIM with P and C containing exactly 2 elements [14]. (Figure 1(e))

We note that in counting both SIMs and MIMs, we ignore self-edges.

We emphasize that we impose the criterion of *maximality *when enumerating SIMs and MIMs. In case of SIM, the set C is maximal, whereas with MIMs both P and C sets are maximal.

These statements define the fundamental network motif set – FFL, SIM, and MIM – as, in a sense, "orthogonal": No subgraph can be more than one of the FFL, SIM, and MIM [13].

## Results

We enumerated the occurrences of FFL, 3-CYC, SIM, MIM, and Bifan subgraph patterns (see Figure 1) in:

1. the transcription networks of *Saccharomyces cerevisiae *(Yeast) under various physiological states [5] (see Table 1(a–f)).

2. the transcription network of *Escherichia coli *[2] (see Table 1(g)), and

3. the signalling pathway of hippocampal CA1 neuron [6] (see Table 1(h)).

**Table 1:** Frequencies of canonical subgraph patterns in biological networks

	FFL	3-CYC	SIM	MIM	Bifan
(a) **Yeast transcription – composite**					

*n*	997	4	107	1551	186
*μ*	993.5	4.2	76.8	1919.2	413.6
*σ*	281.4	2.4	27.0	233.1	111.1
*z*	0.0123	-0.0977	0.6734	-1.5792	-2.0479

(b) **Yeast transcription – Cell Cycle**					

*n*	103	3	27	56	15
*μ*	79.3	1.9	28.0	76.6	31.7
*σ*	22.6	1.3	6.9	11.3	7.2
*z*	1.0491	0.9133	-0.1397	-1.8144	-2.3325

(c) **Yeast transcription – Sporulation**					

*n*	67	2	27	41	26
*μ*	38.0	0.6	30.7	53.0	28.8
*σ*	12.5	0.8	5.1	7.8	7.8
*z*	2.3148	1.7739	-0.7303	-1.5336	-0.3544

(d) **Yeast transcription – Diauxic Shift**					

*n*	64	1	48	137	54
*μ*	63.2	0.3	47.8	141.1	64.4
*σ*	27.2	0.6	13.7	18.2	16.6
*z*	0.0301	1.0626	0.0167	-0.2230	-0.6260

(e) **Yeast transcription – DNA Damage**					

*n*	70	1	45	117	51
*μ*	49.0	0.2	44.9	117.1	53.4
*σ*	25.8	0.5	12.1	17.0	14.4
*z*	0.8149	1.6548	0.0076	-0.0073	-0.1679

(f) **Yeast transcription – Stress Response**					

*n*	42	2	32	46	21
*μ*	36.1	0.3	40.5	52.7	24.0
*σ*	14.2	0.7	9.3	11.7	6.3
*z*	0.4123	2.4005	-0.9102	-0.5698	-0.4761

(g) ***Escherichia coli *transcription**					

*n*	40	0	2	45	17
*μ*	24.1	0.4	4.7	29.0	17.5
*σ*	12.3	0.7	2.8	9.7	5.5
*z*	1.2928	-0.6379	-0.9663	1.6463	-0.1001

(h) **Hippocampal CA1 neuronal signalling pathway**					

*n*	266	37	5	240	92
*μ*	219.3	21.7	4.6	181.1	103.7
*σ*	54.9	6.2	2.1	35.5	14.7
*z*	0.8499	2.4664	0.1994	1.6590	0.7992

Frequencies of FFL, 3-CYC, SIM, MIM, and Bifan in (a-f) various transcription networks of *Saccharomyces cerevisiae*, (g) transcription network of *Escherichia coli*, and (h) signalling pathway of hippocampal CA1 neuron. The observed frequencies, *n*, of these patterns in each of the networks were compared with the corresponding mean frequency (*μ*) in 1000 random networks having same degree sequences. The standard deviation (*σ*), and *z*-score $\left(z=\frac{n-\mu }{\sigma }\right)$ show the statistical relevance of various patterns. Positive and negative values of *z *signify the extent of over- and under-representation respectively, of *n *from *μ *(in *σ *units).

For each network, 1000 random networks were generated conserving the degree sequence of the original network. Comparisons were made between the frequencies of appearances of various patterns in the real network, and the means and dispersions of their appearances in corresponding random networks.

Table 1 presents the significance profiles of various patterns. The results show that the frequencies of various subgraph patterns are *not *significantly over- or under-represented in real networks when compared to their random formulations. A few outliers (where |*z*-score| > 2) appear in Table 1: FFLs in Yeast Sporulation (*z*-score = 2.31), 3-CYCs in Yeast Stress Response (*z*-score = 2.47) and neuronal signalling pathway (*z*-score = 2.4), and Bifans in Yeast Composite (*z*-score = -2.05) and Cell Cycle (*z*-score = -2.33). Some outliers are slightly overrepresented (*z *> 0), and others are slightly underrepresented (*z *< 0). We observe no outliers with |*z*-score| ≥ 2.47.

We employ the same random model as used in earlier related works [2,3,5,7]. While conserving the degree sequence of the original network, the edges in a random network are chosen randomly so that the resultant network is free from the pressure of "evolutionary selection" which is incident on real biological networks. However, in addition to the conservation of the degree sequence, more sophisticated random models can be generated by embedding other connectivity constraints observed in real networks, such as rules of clustering together of nodes in a neighbourhood, and path-lengths between pairs of nodes. *These additional constraints will only make the random null hypothesis more stringent to refute*. Nevertheless, even using the basic random model employed in our work, we fail to gather any statistical evidence that the canonical patterns appear in real networks at non-random frequencies.

We note that there are differences in the counts of various motifs reported by Luscombe et al. [5] and this work, even though we use the same datasets (Table 1(a–f)). Our figures supersede those reported by Luscombe et al. (see [13] for a detailed explanation).

Our reanalysis of *Escherichia coli *transcription network provides the most direct comparison of our results with those of Alon and coworkers (see Table 1(g)). We fail to see any statistical evidence to suggest that the canonical subgraphs appear more frequently than random. On comparing our results with those published by Shen-Orr et al. [2], we find that:

1. Our definitions of fixed size subgraphs such as FFL and 3-CYC are consistent with those originally defined by Alon and colleagues [2,3]. Consequently, we agree on the absolute count of these subgraph patterns in the real network. Surprisingly however, our results of appearances of FFLs in random networks greatly differ. To reconfirm our results reported in Table 1(g), we generated another set of 1000 random networks using an alternative method of random network generation – starting with the original network, over a large number of repetitions, two randomly chosen interactions are swapped. (i.e., interactions: (P1,C1), and (P2,C2) become (P1,C2), and (P2,C1)). Indeed we get similar statistical significance results using this alternative method, compared to those reported in Table 1(g).

2. Our definition of Bifan ensures that we count only those patterns where a pair of target genes are *strictly *regulated by a pair of transcription factors – Bifans are maximal MIMs where P = C = 2. We believe Shen-Orr et al. [2] fail to maintain this strictness, thereby overcounting Bifans by including in their count two parent, two child subMIMs of larger maximal MIMs. (See Discussion.)

3. Similarly, our definitions and enumeration methods of SIMs and MIMs are mathematically more rigorous than those used by Shen-Orr and colleagues [2]. Our counts of maximal MIMs and SIMs could be converted directly to counts of non-maximal MIMs and SIMs (see below). We note therefore that the non-observance of statistically significant differences between natural and randomized networks in counts of maximal MIMs and SIMs *implies *that there are no statistically significant differences between natural and randomized networks in counts of non-maximal MIMs and SIMs. This comment, together with the reminder that our definitions (and counts) of FFLs and 3-CYCs are identical with those of Alon *e*t al., shows clearly that the discrepancies are not a simple effect of alternative definitions of SIMs, MIMs and Bifans.

## Discussion

### The observed discrepancy in occurrence frequency of FFLs and 3-CYCs is a natural consequence of topological properties of networks

Occurrences of FFLs and 3-CYCs in various biological networks (see Table 1) show patterns: there are a relatively large number of FFLs and relatively small number of 3-CYCs. In this section we explain the topological basis for these differences in their frequencies.

First we note that random connectivity within three-node subgraphs itself favours FFLs. Consider a directed, complete – there is an edge between every pair of nodes – three node graph (3-graph). Excluding bidirectional edges, for any set of 3 nodes there are 2^3 ^= 8 possible directed 3-graphs. Each of these configurations is isomorphic to either a FFL or a 3-CYC – any directed complete 3-graph is either a FFL or 3-CYC. Out of 8 possibilities, 6 form FFLs, and 2 form 3-CYCs. Allowing bidirectional edges, there are an extra 19 possible configurations containing at least one bidirectional edge. Each of these possibilities gives multiple FFLs or 3-CYCs or both. With or without bidirectional edges, there is a natural 3:1 bias towards forming an FFL over a 3-CYC in a 3-graph.

Global properties of biological networks also favour FFLs over 3-CYCs. Most biological networks, such as those used in our study, are *scale-free *[15]. In scale-free networks, the connectivity of nodes follows the power law: the probability of a node having *k *neighbours is *P*(*k*) ~ *k*^-*γ*^. Only a few nodes in such a network are highly-connected (and form *hubs*), while most nodes are sparsely connected [15].

We asked how many of the FFLs in various networks contain hubs among their nodes. (We consider as hubs the top 10% of nodes in the network that are highly-connected, having more than 10 neighbours.) Table 2 contains the percentages of FFLs enumerated in various networks, having *n *= {0, 1, 2, 3} nodes as hubs. A large majority of the FFLs contain at least one hub; most common being the FFLs with hubs at two of their nodes. In the Yeast composite network, 961 of 997 FFLs have at least one common *source-intermediate *edge between them. These 961 FFLs can be grouped into 114 clusters (containing distinct source-intermediate edges) revealing that connected hubs often share many common children, automatically giving rise to FFLs. We believe that the principle of *preferential attachment *predisposes a biological network to have connected hubs that have shared children. This gives a network its robustness to random node failure [15].

**Table 2:** Percentage of FFLs in various networks having exactly *n *of its nodes as hubs

	*n *= 1	*n *= 2	*n *= 3	*n *= 0
Yeast Composite	15.7	80.1	2.2	1.9
Yeast Sporulation	22.4	67.2	4.5	6.0
Yeast Cell Cycle	9.7	68.0	15.5	6.8
Yeast Diauxic	12.5	81.2	6.2	0.0
Yeast DNA damage	24.3	68.6	5.7	1.4
Yeast Stress response	21.4	59.5	19.0	0.0
Hippocampal pathway	20.9	58.7	15.5	4.9

We also observe that there is an imbalance between indegree and outdegree around hubs – there are significantly more outgoing edges than incoming edges. We have seen above that FFLs are naturally favoured over 3-CYCs in 3-graphs. The imbalances between in- and out-degree around the hubs further enhances the formation of FFLs. Consider a hub with *m *incoming edges and *n *outgoing edges. With a random addition of an edge between any pair of (*m *+ *n*) nodes adjacent to this hub, the probability of forming an FFL in this system is: $P_{\text{FFL}}=\frac{2{(}^{m}C_{2}{+}^{n}C_{2})+mn}{2{(}^{m}C_{2}{+}^{n}C_{2}+mn)}$ while that of forming a cycle is: $P_{3-\text{CYC}}=\frac{mn}{2{(}^{m}C_{2}{+}^{n}C_{2}+mn)}$. Then, $\frac{P_{\text{FFL}}}{P_{3-\text{CYC}}}=1+\frac{\left(m-1\right)}{n}+\frac{\left(n-1\right)}{m}$, which is symmetric in *m *and *n*. If there is a large disparity between *m *and *n *(i.e., *m *≪ *n*, or *m *≫ *n*), then one of the terms $\left(\frac{m}{n}\right)$ or $\left(\frac{n}{m}\right)$ dominates, resulting in $\frac{P_{\text{FFL}}}{P_{3-\text{CYC}}}\sim \max\left(\left(\frac{m}{n}\right),\left(\frac{n}{m}\right)\right)$. For example, when *m *= 2 and *n *= 20, *P*_FFL _= 0.91, and *P*_3-CYC _= 0.09. This shows the odds against the formation of a 3-CYC in networks with structures typical of biological networks.

There have been suggestions that 3-CYC is an "anti-motif" – a motif that is selected *against *in many biological networks [14]. But, as described above, the suppression of 3-CYCs is an expected consequence of topological properties of biological networks.

*These properties are sufficient to account for the observed profiles of FFLs and 3-CYCs*.

### Assemblies of motifs

**Figure 2:**
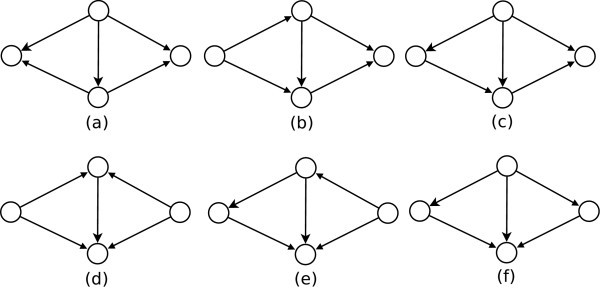
**Self-Assemblies of two FFLs**. Various possible self-assemblies of two FFLs sharing a common edge.

Kashtan and colleagues [16] observed that regulatory networks contain multi-output FFL generalizations (see Figure 2(a)) in frequencies much higher than multi-input (Figure 2(d)) and multi-intermediate (Figure 2(f)) generalisations. (These authors also suggested that multi-output FFLs were selected to achieve some information processing role [16].)

We, in contrast, observe that the varied frequencies of assemblies of multiple FFLs are a consequence of the occurrence of FFLs around hubs. Figure 2 shows all possible assemblies involving two FFLs sharing a common edge. In Table 3 we enumerate the occurrences of each such assembly in various networks. Clearly, the multi-output assembly of two FFLs abounds over other possibilities, simply because a large number of FFLs share a common source-intermediate edge.

**Table 3:** Number of occurrences of various assemblies shown in Figure 2

	Frequencies of patterns in Figure 2					
	(a)	(b)	(c)	(d)	(e)	(f)
Yeast Composite	9232	259	184	288	280	152
Yeast Sporulation	113	3	8	21	8	4
Yeast Cell Cycle	419	22	17	38	12	15
Yeast Diauxic Shift	214	2	2	3	4	5
Yeast DNA damage	140	6	6	11	4	8
Yeast Stress Response	41	9	6	5	4	1

**Figure 3:**
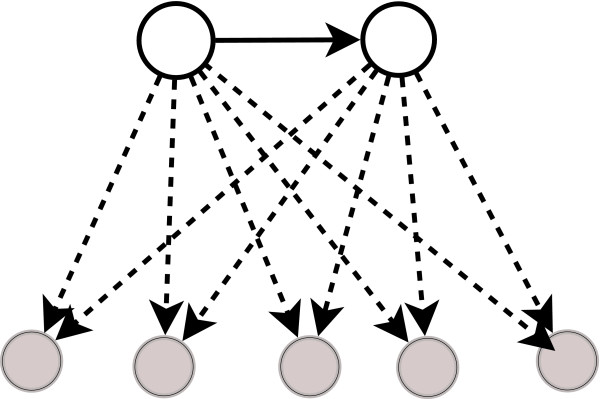
**Example of FFLs sharing two hub nodes**. Example of FFLs sharing two hub nodes that are connected.

Thus the numbers of multi-output FFLs grow combinatorially with the number of FFLs sharing a common source-intermediate edge. The count of (*k*<*n*)-output assembly of FFLs, where *n *is the number of FFLs sharing two common (source and intermediate) nodes, is expected to increase as ^*n*^*C*_*k*_. For example, 5 FFLs having a common source-intermediate edge (see Figure 3) will give rise to 10 non-redundant bi-output FFLs. Table 4 shows the statistical significance of finding bi-ouput FFLs in various real networks used in this work, by comparing the occurrences with those observed in their corresponding random networks. Statistically, their frequencies are not significantly greater than in random networks.

**Table 4:** Frequencies of Bi-FFL assembly in various networks

	*n*	*μ*	*σ*	*z*
Yeast Composite	9232	17278.2	13537.5	-0.6
Yeast Sporulation	113	52.4	48.1	1.3
Yeast Cell Cycle	419	173.8	132.2	1.9
Yeast Diauxic Shift	214	238.4	334.3	-0.1
Yeast DNA Damage	140	189.6	295.8	-0.2
Yeast Stress Response	41	67.2	69.3	-0.4
Ecoli transcription	0	0.6	1.1	-0.6
Hippocampal pathway	85	327.0	223.6	-1.1

See Table 1 legend for explanation of symbols.

### On SIMs, MIMs and Bifans

SIMs and MIMs are variable sized subgraphs. Alon and colleagues [2] defined the dense overlapping regulon (DOR) as a two-layered subgraph with *not necessarily complete *connections between them. MIMs are special DORs, a concept that arose as a generalization of the Bifan (Figure 1(e)) subgraph. These Bifans were observed to be present in large numbers in biological networks. However, some investigators fail to impose the criterion of maximality while counting MIMs. This can lead to significant inflation of counts [2,5]. *Note that this applies equally to natural graphs and random ones* (Hence we emphasize that the differences between our results and those of Alon et al. are not explicable solely on the basis of alternative definitions of some of the motifs).

A maximal MIM with *m *parents and *n *children contains [2^*m *^- (*m *+ 1)] × [2^*n *^- (*n *+ 1)] - 1 easily enumerable non-maximal "subMIMs". Our definition of a Bifan ensures that we are only counting (maximal) MIMs that contain 2 parents and 2 children. Counting subMIMs as Bifans will combinatorially increase their counts, as each maximal MIM will contribute to ^*m*^*C*_2 _× ^*n*^*C*_2 _Bifans. For example, the Yeast composite network contains a large MIM containing 2 parents and 119 children. This alone contributes to 7021 non-maximal Bifans. The same consistency is maintained when counting SIMs. The list of subgraphs occurrences in various networks used in this paper can be downloaded from .

The natural appearance of bipartite graphs in dense general graphs has received some attention in graph theory [17]. It has also been demonstrated, using Ramsey theory [18], that bipartite cliques appear in sufficiently dense bipartite graphs [19,20]. MIMs are bipartite cliques. Biological networks contain regions in which dense bipartite graphs naturally appear, and hence giving rise to bipartite cliques. This in itself speaks against the notion of evolutionary selection of MIMs [2].

### Evidence for selection of motifs?

Analysis of natural networks shows that several commonly observed subgraphs identified as motifs do not appear at frequencies significantly greater than in corresponding random graphs. Instead, their frequency of occurrence is the result of the small-world character of many biological networks, and of the associated degree distribution.

What does this imply about the idea that motifs have been selected, by evolution, for function? The statement that motifs are selected for function has two possible interpretations, not necessarily incompatible:

1. It might be asserted that the *general type *of motif – for instance FFL rather than 3-cycle – is selected because of a general propriety to serve a particular function (For example, Alon et al. [1] pointed out that a FFL with AND logic at the output node can function as a filter rejecting transient stimuli).

2. Or it might be asserted that *individual *FFLs (or 3-cycles) within a network play specific functional roles at specific points.

Statistics of frequency of occurrences of specific motifs, and the comparison of observed frequencies in natural networks relative to random networks, do not – no matter what numerical results emerge – provide evidence for or against assertions of type 2. If any individual subgraph at some node plays an essential functional role in a network, it could be selected – whether it is a commonly-occurring subgraph or not. Conversely, an observation of significantly non-random occurrence frequencies of motifs would suggest the action of positive or negative selection, acting at the level of assertions of type 1 or type 2. Indeed it seems inescapable that if assertions of type 1 are true, then at least some assertions of type 2 must also be true, but not vice versa.

Our results suggest that there is no evidence for type 1 assertions.

## Conclusion

We have analysed several biological networks. Our results suggest that there is no evidence suggesting selection for or against subgraph patterns such as FFL, 3-CYC, SIM, MIM, Bifan. We have shown that, in contrast to the need to invoke selection to explain the structure of observed networks, it is the topological properties of networks that automatically favour the observed frequency profiles of various subgraph patterns.

## Authors' contributions

Both the authors contributed equally to the planning and execution of this study; both authors contributed to the draft, and have read and approved the final manuscript.
